# “*We are like co-wives*”: Traditional healers' views on collaborating with the formal Child and Adolescent Mental Health System in Uganda

**DOI:** 10.1186/s12913-018-3063-4

**Published:** 2018-04-10

**Authors:** Angela Akol, Karen Marie Moland, Juliet N. Babirye, Ingunn Marie S. Engebretsen

**Affiliations:** 1 Center for International Health, University of Bergen, Bergen, Norway; 2 Makerere University School of Public Health, Kampala, Uganda

**Keywords:** Traditional healers, Mental health, Child and adolescent, Health system

## Abstract

**Background:**

Early identification and management of mental illness in childhood and adolescence helps to avert debilitating mental illness in adulthood but the attention given to Child and Adolescent Mental Health (CAMH) has until recently been low. Traditional healers are often consulted by patients with mental illness and in Uganda, up to 60% of patients attending traditional healers have moderate to severe mental illness. Poor access to CAMH care in Uganda creates a treatment gap that could be met through enhanced collaboration between traditional healers and biomedical health systems. The aim of this study was to explore traditional healers’ views on their collaboration with biomedical health systems so as to inform the implementation of strategies to improve access to CAMH services in Uganda.

**Methods:**

In-depth interviews with 20 purposively selected traditional healers were conducted in November 2015. A semi-structured interview guide was used to explore: 1) The experiences of traditional healers with mental ill-health in children and adolescents; 2) their willingness to collaborate with the formal health system; and 3) their perception of clinicians’ willingness to collaborate with them. Interviews were conducted in local languages and tape recorded. Data were analysed using thematic analysis.

**Results:**

Traditional healers described several experiences managing children and adolescents with mental illness, which they ascribed to spiritual and physical causes. The spiritual explanations were a consequence of unhappy ancestral spirits, modern religions and witchcraft, while physical causes mentioned included substance abuse and fevers. No traditional healer had received a patient referred to them from a medical clinic although all had referred patients to clinics for non-mental health reasons.

Traditional healers expressed distrust in biomedical health systems and believed their treatments were superior to medical therapies in alleviating mental suffering. They expressed willingness to collaborate with biomedical providers. However, traditional healers believe clinicians disregard them and would not be willing to collaborate with them.

**Conclusion:**

Potential for collaboration between traditional healers and biomedical health systems for improving access to CAMH services in Uganda exists, but is undermined by mutual mistrust and competition between traditional healers and clinicians.

**Electronic supplementary material:**

The online version of this article (10.1186/s12913-018-3063-4) contains supplementary material, which is available to authorized users.

## Background

Early identification and management of mental illness in childhood and adolescence is important for averting debilitating mental illness in adulthood [1]. Child and Adolescent Mental Health (CAMH) refers to a range of mental, neurological and substance use (MNS) disorders that occur in childhood and adolescence. Mental health has been a neglected global health area and the attention given to CAMH has until recently been disproportionately low, compared to mental disorders in adults and the elderly [2]. As a result, a huge treatment gap for CAMH conditions persists, one that could be addressed by improving access to CAMH care in LMIC [3].

The importance of access to mental health services for all is accentuated by the UN Sustainable Development Goals (SDGs), which acknowledge mental health as a development priority for which service coverage indicators are imperative [4]. The inclusion of mental health in the SDGs came in the wake of global discussions in the last decade on eliminating barriers to equitable care [5–7], a position adopted by the World Health Organization (WHO) in 2001 [2].

Access to mental health services is important for Primary Health Care (PHC) [2]. This opinion is reinforced by the 2008 World Health Report which revisited the Alma Ata goals and emphasised the need for engaging culturally competent providers who respect patient beliefs [8]. By providing care in line with indigenous knowledge and belief systems, traditional healers fit this description [9, 10]. Two WHO strategies currently endorse the involvement of traditional healers in care: the WHO’s traditional and complementary medicine strategy 2014–2023 which highlights traditional healers as a potential solution to achieving universal health coverage [11]; and the WHO mental health action plan 2013–2020 which encourages greater collaboration with traditional healers to promote mental wellbeing [12]. However, there are no clear examples of collaboration between traditional healers and the biomedical health system for mental health care.

Collaboration between traditional healers and clinicians in alleviating mental suffering among children and adolescents is particularly important in settings where access to CAMH services is poor, such as Uganda, which has fewer psychiatric facilities than the global average [13]. However, hindrances to successful collaboration between traditional healers and biomedical mental health systems are cited in qualitative studies. For instance, traditional healers express a preference for referring clients to another healer rather than to a clinician, maintaining an ‘internal’ referral network. Other traditional healers express skepticism regarding the value of biomedical psychiatric treatments because of their perceptions of the underlying spiritual cause of mental disorders [14]. Conversely, biomedical service providers think that the difficulty around establishing the scientific validity of traditional and faith healers’ practices makes referral to traditional healers very difficult [15]. These attitudes reflect a lack of trust between traditional healers and biomedical providers. Yet trust drives cooperation between agents of the health system; without it, collaboration breaks down, corroding the interactive nature of health systems [16].

**Fig. 1:**
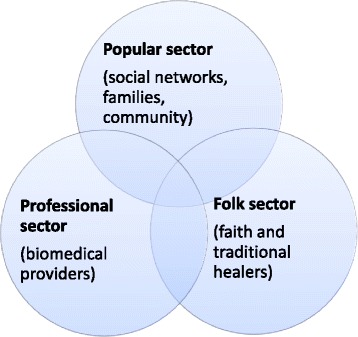
Three sectors of health care, adapted from Kleinmann A (1980)

The interaction between the different agents of health care is embodied by the classical Explanatory Models of Illness theory proposed by Kleinman and others. Under this model, patient satisfaction with care is more likely with providers who explore and address patients’ explanatory models of illness [17]. Thus, health care is composed of different sectors each with its unique institutions and each relying on different patient explanatory models (Fig. 1). The model states that while the professional / biomedical sector is one sought by patients who ascribe illness to biological causes, the folk and popular sectors tend to attract clients who attach a social or a cultural explanation to illness. Each of the sectors offers unique remedies, related both to the expertise of providers within the sector and to the perceived cause of illness. Clients may utilise a combination of two or more sectors for a single episode of illness and therefore, an interaction between the sectors is required to achieve access to and satisfaction with care [18].

Estimates indicate that more than 80% of African populations attend traditional healers for health reasons and that 40–60% of these have some kind of mental illness [11, 19]. In fact, traditional healers are the first recourse for approximately half of individuals seeking care for mental disorders in Africa [20]. In Uganda, available research indicates that up to 60% of Ugandan patients attending traditional healers’ shrines have moderate to severe mental illness [21]. Several studies have documented positive outcomes among adults of mental health care from traditional healers in sub Saharan Africa [21–23], a factor which could explain traditional healer popularity. The widespread use of traditional healers could also be attributed to the accessibility of traditional healers relative to medical professionals; across sub Saharan Africa, there is a traditional healer for every 500 people, compared to a doctor: population ratio of 1: 40,000 [24]. While it has not been possible to determine the total number of traditional healers in Uganda, estimates indicate that compared to a doctor: population ratio of 1:8547, there is one traditional healer for 700 Ugandans [25].

Traditional healers are widely consulted worldwide for treatment of various ailments and practice traditional medicine, which is defined as knowledge and remedies based on beliefs indigenous to different cultures used in the prevention, diagnosis or management of physical and mental illness [11]. For purposes of this paper, traditional medicine refers to such practices applied with the aim of diagnosing, treating or otherwise alleviating mental suffering among children and adolescents. In this paper, the term ‘bio medical personnel’ is used interchangeably with ‘clinicians’. The aim of this study was to explore traditional healers’ views on their collaboration with biomedical health systems so as to inform the implementation of strategies to improve access to CAMH services in Uganda. Specifically, we aimed to examine the experiences of traditional healers with CAMH; their perception of and attitude towards collaboration with biomedical mental health systems.

## Methods

### Design

This qualitative study employed semi-structured interviews with 20 purposively selected traditional healers, held in November 2015. Twenty was the number of participants designated before the onset of the study, based on local estimates of traditional healers with CAMH experience. Twenty was also deemed an appropriate sample size for achieving saturation [26] while preserving validity. Study participants were selected by the leader of the traditional healers in the Eastern region of Uganda, based on his knowledge of traditional healers engaged in mental health treatment. The traditional healers ranged in age from 26 to 80, with a mean age of 53. Six of them were female, and the median years of practice was 23 (range 6–52). All except one traditional healer had directly managed children and adolescents with mental illness, with 11 as the mean number of reported patients.

An interview guide (Additional file 1) developed by the principal investigator (PI) was used to explore: 1) The experiences of traditional healers with mental ill-health in children and adolescents; 2) Their willingness to collaborate with biomedical health systems; and 3) their perceptions about the willingness of clinicians to collaborate with traditional healers. Semi-structured interviews were selected as the method of choice because of their ability to elicit rich descriptions of individual experiences (31).

### Data collection

Interviews were conducted in local languages (Lumasaaba and Luganda) by a research assistant with a sociology background, experienced in qualitative research. The research assistant was selected based on residence in the area, fluency in the two local languages and experience conducting research with traditional healers. Prior to conducting the interviews, the PI trained the research assistant on the main objectives of the study and the study tool. The PI did not participate in the interviews due to language limitations and because we believed that the presence of a medical doctor might inhibit the traditional healers’ responses and restrict access to their premises.

All interviews were tape recorded. Interviews lasted approximately 45 min and were held at a location convenient to the traditional healer, mostly at their homes and workplaces.

### Data analysis

Interviews were transcribed directly into English by the same research assistant who conducted the interviews, while taking care to preserve important local language concepts. Data were analysed using thematic analysis. In keeping with this methodology, the transcripts were read and re-read to obtain immersion and a good understanding of the data.

Transcripts were imported into QSR NVivo 11 *(QSR International Pty Ltd. Version 11, 2015).* Open coding was then conducted by a single coder to categorise data and organise it into themes. An iterative process of labelling and extraction of data into themes and sub-themes, comparing and contrasting them with other themes followed. Coding and analysis were led by the lead author (AA), a medical doctor with broad experience of the public health system. The last author (IMSE) reviewed all interview transcripts.

### Ethical considerations

The study received approval from the Makerere School of Public Health Higher Degrees Research and Ethics Committee and from the Uganda National Council for Science and Technology. All ethical guidelines involving human subjects were adhered to throughout this study. All interviews were tape recorded after obtaining participants’ consent.

## Results

Two main themes arise from the data: 1) Treating mental illness is cultural; and 2) Mistrust hampers collaboration. The theme “Treating mental illness is cultural” presents data on epistemologies of mental illness in children and adolescents; and on interactions between traditional healers and biomedical providers. “Mistrust hampers collaboration” is categorized into two sub-themes: willingness to collaborate and barriers to collaboration.

### Treating mental illness – A culturally founded skill

Traditional healers’ narrative on their experiences treating mental illness focused on: explanations for mental suffering in children and adolescents and accounts of their interactions with biomedical health systems. The two issues are closely interlinked. As will be illustrated in this section, different explanations of illness based on different epistemologies resulted in treatment regimes that determined the nature of interaction with the formal health system.

#### Mixed explanations of mental illness

*“What I know is that some of the mental health problems in children and young people is caused by ancestral clan spirits, especially if these spirits want the person initiated into being a traditional healer and the person resists… and not until this person is initiated into traditional healing his mental disorder never heals”* – Traditional healer 13, twenty-seven years’ practice

In keeping with traditional healers’ belief in ancestors’ ability to interfere in the lives of the living, ancestral spirits played a major role in explaining mental illness. All traditional healers cited unhappy ancestral spirits as a cause of mental ill-health among children and adolescents. A commonly held view was that ancestral spirits were unhappy because ancient customs and rituals have been abandoned; and that children and adolescents who resisted their destiny to become traditional healers inevitably developed mental illness, which was only curable by initiation into traditional healing.

We also found prevalent perceptions of mental suffering being consequent to conflict between traditional customs and modern ‘born again’ religions, as illustrated by this excerpt *“…it happens very much especially to those people who have abandoned issues with traditions and opted for the religion of born again…”* This conflict between traditional and modern ‘born again’ religions was considered responsible in particular, for protracted mental ill-health among children and adolescents. Other causes mentioned by all traditional healers were ghosts, spirits and witchcraft, which are sent by enemies and encountered by people who walk outside the house at night, causing them to descend into mental illness. Prosperous families were considered particularly prone to witchcraft from jealous people, leading to mental illness among children.

Traditional healers also ascribed mental ill-health to non-spiritual and non-social causes. All traditional healers implicated substance abuse as a cause of mental illness among adolescents. Substances mentioned were a local potent brew, *waragi*; narcotic drugs - *enjaga*; tobacco and aviation fuel, taken singly or in combination. Most also cited high fever and cerebral malaria as a cause of mental disturbances in children. According to them, this category of mental illness was best treated in hospitals and clinics.

A common view was one of unsanitary conditions at birth and early childhood leading to mental illness. The pathway through which such conditions were believed to cause mental disease is through breathing difficulty, as illustrated by this elderly traditional healer with 45 years of practice, *“I know that if a baby is born in a dirty environment, or…if a child’s head is not protected from the cold air…that child automatically gets a mental disorder when he grows up…the child’s brain is affected directly…begins by having difficulties in breathing…with time this child gets worse and then one realises that a mental disorder has set in”* .

Worms and maggots growing in the child’s brain, were also widely implicated, as explained by the elderly traditional healer: *“once a child starts getting difficulty in breathing it means that he has a maggot in his brain…as the child grows the maggot also grows…this can bring about terrible mental disorder.”* We found that many of the treatments described by the traditional healers were aimed at expelling these maggots; unless the maggot was expelled, the patient would not get well.

*“…usually those who have a maggot in the brain, when it moves, they become very violent … but once the maggot comes out then that person gets completely healed… I remember very well was a 14-year-old girl, who was brought to my place when very violent. So, what I did, I mixed herbs and I poured it through her nose… later she sneezed and two maggots popped out of her nose…”* Traditional healer 09, eleven years’ practice.

In summary, three types of explanations for mental illness were found to be part of the epistemology of the traditional healer: Spiritual explanations including ancestors and neo-Pentecostal worship; social explanations including witchcraft and evil-eye, and physical or natural agents like maggots, infections and substance abuse.

#### No interaction with the formal health system

*“…nobody should deceive you that mental illness can be managed by hospitals….”* Traditional healer 04, eight years’ practice

Traditional healers’ interaction with clinicians was characterised by views about referral to and from health clinics, and by opinions about the competence of clinical practitioners. We found all traditional healers believed that traditional medicine is the only effective treatment for mental ill-health, due to the spiritual nature of the condition. Several traditional healers cited the inability of clinical providers to expel maggots from patients’ brains.

We found very little experience of referral from health clinics to traditional healers. Two participants reported such referral for mental ill-health, after repeated treatments at the medical clinics had failed to make them better:

*“There is one …in the main hospital who one time directed a man with his son to me for management, I hear they had gone to the hospital several times but the boy never got well… I worked on him and he became okay…”-* Traditional healer 17, twenty years’ practice.

However, self-referrals were commonly reported, in which patients discharged themselves from health clinics to consult traditional healers.

Although biomedicine was considered limited in approach, addressing only the physical causes of disease, all informants had referred patients to biomedical clinics. The commonly cited reasons for referral were for rehydration, or for blood transfusion. Others referred patients whom they deemed to have biomedical conditions, particularly malaria, which they were not well suited to manage. One older, more experienced traditional healer mentioned that it was his policy to treat a patient thrice only, following which he would refer to medical clinics. However, such referrals were reportedly not well received by clinicians, if it was known that the patient had consulted a traditional healer:

*“One time I referred a child to Mbale Hospital after I had smeared herbs on the child. On arrival, the doctors chased the patient away accusing them of being dirty…I always send patients to them for management, but for them they have never done so.”* Traditional healer 10, ten years’ practice

Despite great skepticism of the effectiveness of biomedicine on mental illness, some traditional healers integrate biomedical elements into their mental health treatment regime. One example is the traditional healer who professed to routine use of largactil® on violent patients who were brought to him, prior to administering his herbal treatments:

*“…I love using it because it really puts a person to sleep…I’m a traditional healer but I have found out that [largactil®] is a very effective drug when it comes to calming down the person with mental health disorders especially when they are violent...”* – Traditional healer 02, forty-five years’ practice

The view that clinical practitioners are not competent to manage mental health conditions was unanimous. The reasons cited were that clinical practitioners do not comprehend spiritual matters and are poorly placed to treat conditions with a spiritual origin. It was widely acknowledged that they could manage conditions that arose from malaria and other fevers. To prove this point, many of the traditional healers cited examples of patients who had been repeatedly treated at health clinics but only got better after visiting traditional healers. According to the traditional healers, the remedies provided in clinics are temporary; the only lasting effect was believed to come from traditional healers.

### Mistrust hampers collaboration

*“I don’t see it happening easily because those doctors despise all our work. They regard it as satanic and dirty” –* Traditional healer 09, eleven years’ practice.

Even if nearly all traditional healers expressed willingness to collaborate with clinicians in alleviating mental suffering in children and adolescents, their willingness was conditional on clinicians’ reciprocating this goodwill, which was considered unlikely. All the participants believed that clinical providers are not willing to collaborate with traditional healers as they consider them dirty, unsanitary and of a lower education status:

*“You know they regard us as …illiterate and of low class…they regard themselves as people of high class…” –* Traditional healer 02, forty-five years’ practice

Different from their views on clinicians, we found the traditional healers unanimous in their conviction that patients would welcome their collaboration with the formal health system. According to the traditional healers, all patients needed was to get well, so it did not matter through which means they received treatment. They also argued that patients would cease to consult them in secrecy once collaboration was implemented.

*“I see that they will be happy for the collaboration because they will no longer come to the traditional healers in hiding as they do now. They will consult us openly as they do with the clinics” –* Traditional healer 12, ten years’ practice.

We found several perceived barriers to collaboration among the traditional healers. Some of the barriers such as the competence of peers were intrinsic to the traditional healers themselves. Traditional healers viewed their peers who are not ‘specialized’ in mental illness as largely being incompetent for handling CAMH and mental ill-health in general. Advertisement in news media was viewed as a sign of incompetence. It was widely held that competent traditional healers need no advertisement to enhance their reputation; Competence was thought to increase with experience and years of practice.

*“What I would like to tell you is that a real traditional healer does not advertise him / herself over the radio or TV, so once you see one doing this, then know that this person is incompetent in his work…you know there is a lot of joblessness in Uganda, so we have so many who call themselves that they are traditional healers, when they are not, they are simply looking money so that they are able to put food on the table.” –* Traditional healer 07, thirty-four years’ practice.

Another intrinsic barrier perceived by the traditional healers is their lack of English language knowledge. According to the traditional healers, clinical providers would use English language as a means for excluding the less educated traditional healers

*“The barrier I foresee…our counterparts the doctors want always to use English so as to push us away…I see that as a problem” –* Traditional healer 07, thirty-four years’ practice.

We found that traditional healers did not trust biomedical practitioners. In addition to the belief that medical providers viewed them negatively, most traditional healers thought that clinicians would extract knowledge from the traditional healers and use it for their own credit.

*“Working with them is not easy because they don’t like us at all, we are like co-wives who don’t like each other and share one man…” –* Traditional healer 18, fifty years’ practice

*“What I see is that the formal health worker will only take our ideas and use them, therefore, this will only benefit them by them getting more money and traditional healers will not benefit at all.” –* Traditional healer 13, twenty-seven years’ practice

To eliminate barriers, necessary conditions for collaboration were described. Most of the traditional healers mentioned the government as needing to take a lead in integrating them with formal health systems, without which collaboration wouldn’t be possible. The required government intervention mostly suggested was a law or policy recognising traditional healers and compelling clinicians to collaborate with traditional healers;

*“Once government makes a policy for us to be recognized as formal health workers things will just fall in place”-* Traditional healer 15, thirty-seven years’ practice

*“…If a law is put in place then they will accept.”-* Traditional healer 6, fourteen years’ practice

Alongside laws and policies, increased recognition by government, sensitisation of communities, traditional healers and medical providers was cited as a necessary condition for successful collaboration.

## Discussion

The main findings of the study were: 1) Epistemologies of mental illness in children and adolescents are shared by traditional healers; 2) traditional healers have limited interactions with the biomedical health system for mental illness; and 3) traditional healers’ willingness to collaborate with biomedical providers is hampered by mistrust. Whereas previous studies [21, 27] have addressed traditional healers’ views on help-seeking behaviour and have characterised mental health conditions and outcomes, this is the first study in Uganda that has explored the views of CAMH-experienced traditional healers on their integration with formal health systems.

In line with the classical Explanatory Models theory proposed by Kleinman, the epistemological view of mental illness among traditional healers was tinged by their belief systems. Specifically, their reliance on spiritual and supernatural explanations of mental disorders is in line with their identity as traditional healers. This has been found in other studies as well [28]. Nevertheless, some causes of CAMH illness they identified, namely substance abuse and fevers, are shared by biomedical practitioners, illustrating the intersection between the traditional and professional sectors of CAMH care.

That traditional healers are engaged in managing CAMH cases is unsurprising and has been documented by other studies in Uganda and elsewhere [19, 29, 30]. What hasn’t been widely reported, however, is the cross-over between biomedical and traditional treatments, exemplified in the present study by the traditional healer who routinely uses largactil®, a brand name for chlorpromazine. The use of biomedical remedies by traditional healers reinforces the notion that epistemologies are not clear cut – one sector of health care may borrow technologies from another. It also further highlights Kleinman’s conceptual overlap between the professional and folk sectors of care. As suggested by Kleinman therefore, this overlap is one that can be explored in enhancing collaboration between the two sectors.

As in this study, Van Niekerk et al., (2014) reported a low level of referral from clinical practitioners to traditional healers in South Africa. Likewise, Musyimi et al., (2016) found that traditional healers in Kenya had no experience of referral from clinical practitioners [15, 31]. In keeping with Kleinman’s theory [17], the most prevalent referrals found are self- referrals. Patients appear to use the traditional and biomedical sectors interchangeably, possibly depending on factors that may include access and illness interpretation.

The low levels of referral between traditional healers and clinicians could be related to the lack of mutual trust between traditional healers and clinical practitioners found in this study. As noted, trust is a prerequisite for successful collaboration between health care managers and practitioners [16, 32, 33]. In this study, the mistrust is limited to mental health conditions, exemplified by the non-mental health referrals being made. Lack of trust for mental health services has been found elsewhere [15, 34], and is justified by recent findings from sub Saharan Africa that confirm clinicians’ perception of traditional healers as dirty and unprofessional [15, 34, 35]. Suggested ways of addressing this negativity is creating means for dialogue between traditional healers and clinical providers, which has been successful in Kenya (30). Specifically, the health system needs to address the negativity with which patients referred from traditional healers are received at biomedical clinics. Trust of other traditional healers is also poor, and may seem surprising until one considers the high mean age of 53 in this sample; older, more experienced traditional healers whose reputation is based on years of practice could be wary of younger healers who advertise their services and charge exorbitantly for their services, jeopardizing the quality of their occupation. However, this finding suggests too that traditional healers are not homogenous, and potential for competition exists alongside potential for collaboration.

In this study, traditional healers believe themselves to be superior to biomedical providers in treating CAMH disorders, as biomedical providers possess no understanding of spiritual matters. This finding is in sharp contrast to their belief that clinicians regard them as inferior; and suggests that adoption of biomedical explanatory models, treatments and remedies by traditional healers is as unlikely as the adoption of traditional models by clinicians might be. At first look this would appear to raise a conflict that would constrain collaboration. However, viewed in light of the different sub-sectors of health care suggested by Kleinman [17, 18], this finding reinforces the need for a collaborative model, in which the relative competencies of the different sub sectors are recognized and respected.

Traditional healers perceive their devaluation by biomedical providers as a hindrance to collaboration. On the other hand, traditional healers also tend to devalue doctors’ CAMH epistemologies, even as they borrow certain treatments from the biomedical sector. This suggests that traditional healers, whilst professing superior knowledge, find value in biomedical sector therapies. This contradiction is one that appears to mask a willingness to collaborate more with biomedical providers. In fact, willingness to collaborate has been documented elsewhere [15, 34, 35]. The fact that willingness in this study is conditional on traditional healers’ contributions not being overshadowed by the clinical providers suggests strongly that an element of competition exists between the two categories of provider. Competition is counter to the Explanatory Models of Illness theory which is more collaborative than competitive. It is worth noting that whereas the traditional healers’ attitude to biomedical epistemologies is dismissive, clinicians are perceived as less of a threat than ‘neo-Pentecostalism’.

The results of this study have several implications for improving access to CAMH services through traditional healers’ collaboration with clinical mental health systems. First, advocates for collaboration should consider the different explanatory models existent among the two categories of provider; and find mutually acceptable ways to stimulate collaboration, recognising that the two categories have a complementary role to play in stimulating access to CAMH services. This should involve, among others, improving clinicians’ acceptance of traditional healers’ explanatory models for illness; and vice versa. Secondly, trust between the two categories of provider needs to be enhanced to improve interaction between the two sectors, which currently operate in isolation. In particular, the perceived negativity by clinicians of traditional healers needs to be addressed. Thirdly, quality of care by traditional healers needs to be addressed to improve hygiene and eliminate unethical practices like extortion of money from clients. These could form suggestions for inclusion in policy suggested by traditional healers to guide collaboration between the two sectors of care.

Some strengths of this study are recognised. The use of a purposive sample of traditional healers with CAMH experiences provides experience-based views. Thus, we believe that these views are transferable to all traditional healers with CAMH experience in Uganda considering that the health system and policy context is uniform across the country. Also, given that traditional healers believe they are disliked by clinicians, conducting interviews at traditional healers’ place of work and the use of a non-medical interviewer probably improved the traditional healers’ ability to express their views freely. As limitations, we note that clinicians’ views were not sought to validate traditional healers’ impressions; however, the aim of this study was to explore CAMH services from the traditional healers’ perspective. Secondly, we recognize that the reliance on a single coder, and the direct translation of interviews into English during transcription could have introduced a bias resulting from the coder’s and transcriber’s own interpretation of the data; and threatened linguistic equivalence since back-translation of local language transcripts was not done. Thirdly, the absence of the PI at interviews might have limited deeper enquiry into the subject matter as the data were collected.

## Conclusion

These findings show that potential for collaboration between traditional healers and biomedical health systems for improving access to CAMH in Uganda exists, based on shared epistemologies and technologies; and patients’ movement across the two sectors. However this potential is undermined by the prevailing mutual mistrust and competition between traditional healers and clinicians. The findings highlight the need for implementation of strategies that harness the complementarity of traditional and biomedical sectors of mental health care as a means of improving access to CAMH services.

## Additional file

Additional file 1: In-depth Interview guide: A semi-structured question guide to facilitate interviews with traditional healers. (DOCX 16 kb)

## Abbreviations

CAMH: Child and Adolescent Mental Health
LMIC: Low- and Middle-Income Countries
MNS: Mental, Neurological and Substance Use
PHC: Primary Health Care
PI: Principal Investigator
THETA: Traditional and Modern Health Practitioners Together Against AIDS
WHO: World Health Organisation
