# Supplementary material for: “We are like co-wives”: Traditional healers' views on collaborating with the formal Child and Adolescent Mental Health System in Uganda
Source: BMC Health Serv Res. 2018 Apr 10;18:258. doi: 10.1186/s12913-018-3063-4 (PMC5892042; doi:10.1186/s12913-018-3063-4)
Supplement: Supplementary file 1 — In-depth Interview guide: A semi-structured question guide to facilitate interviews with traditional healers. (DOCX 16 kb) [file 12913_2018_3063_MOESM1_ESM.docx]

**CAMH Integration study in Uganda**

**In-depth Interview Guide**

**Name of Interviewer____________________________________________________**

**Date ________________________________________________________________**

**Name of Interviewee____________________________________________________**

This guide is designed for interviews with traditional healers prior to implementation of an integration pilot

Good morning. I am ________ (introduce self).

This interview is being conducted to get your input about engagement of traditional healers in Child and Adolescent Mental Health. I am especially interested in your attitudes, concerns and perspectives on the engaging with traditional healers in the care of children and adolescents with mental health disorders.

If it is acceptable to you, I will be tape recording our conversation. The purpose of this is so that I can get all the details but at the same time be able to carry on an attentive conversation with you. I assure you that all your comments will remain confidential. I will be compiling a report which will contain your comments without any reference to individuals. Do you agree to this interview and the tape recording?

I'm now going to ask you some questions that I would like you to answer to the best of your ability. If you do not know the answer, please say so.

1. I'd like to start by having you briefly describe your perceptions of mental health problems
   1. What do you believe leads to mental disorders in children and adolescents?
   2. How much experience do you have managing children and adolescents with mental health disorders?
   3. In your opinion how competent are THs with dealing with CAMH?
2. What is your perception of the formal health system?
   1. Describe your engagements with the mental health system in the past
   2. Please comment on the effectiveness of formal health systems in handling CAMH disorders
3. I would now like to ask about traditional healers working with the formal health system for treatment of child and adolescent mental health
   1. How willing would you and your colleagues be to identify children with mental health disorders and refer them to the formal health system for care *(probe to gather information on acceptability and reasons for chosen stance)*
   2. What effect would such an intervention have on the sense of empowerment among traditional healers?
   3. What barriers to successful integration do you foresee?
   4. What facilitators are necessary to be in place for a successful integration initiative?
   5. Which government and traditional healer structures need to be consulted?
4. How do you rate patients’ acceptance of integration?
5. How do you rate health workers’ acceptance of integration?
6. Is there any other information that you think would be useful for me to know?
